# *N*-*p*-Tolyladamantane-1-carboxamide

**DOI:** 10.1107/S1600536809036101

**Published:** 2009-09-12

**Authors:** Weiwei SiMa

**Affiliations:** a Ordered Matter Science Research Center, College of Chemistry and Chemical Engineering, Southeast University, Nanjing 210096, People’s Republic of China

## Abstract

In the crystal of the title compound, C_18_H_23_NO, the mol­ecules are linked into chains along the *c* axis by inter­molecular N—H⋯O hydrogen bonds.

## Related literature

For For bond-length data, see: Allen *et al.* (1987 ▶). For the synthesis of the title compound, see: Karle *et al.* (1997 ▶); Tadashi *et al.* (1969 ▶)
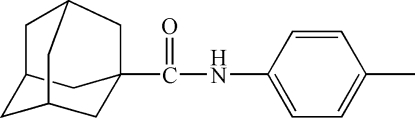


## Experimental

### 

#### Crystal data

C_18_H_23_NO
*M* *_r_* = 269.37
Orthorhombic, 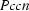

*a* = 30.708 (7) Å
*b* = 9.7927 (2) Å
*c* = 10.0203 (6) Å
*V* = 3013.2 (7) Å^3^
*Z* = 8
Mo *K*α radiation
μ = 0.07 mm^−1^
*T* = 298 K
0.50 × 0.30 × 0.30 mm

#### Data collection

Rigaku SCXmini diffractometer
Absorption correction: multi-scan (*CrystalClear*; Rigaku, 2005 ▶) *T*_min_ = 0.964, *T*_max_ = 0.978
27405 measured reflections
3443 independent reflections
2652 reflections with *I* > 2σ(*I*)
*R*_int_ = 0.080

#### Refinement

*R*[*F* ^2^ > 2σ(*F* ^2^)] = 0.073
*wR*(*F* ^2^) = 0.174
*S* = 1.16
3443 reflections
182 parameters
H-atom parameters constrained
Δρ_max_ = 0.20 e Å^−3^
Δρ_min_ = −0.15 e Å^−3^

### 

Data collection: *CrystalClear* (Rigaku, 2005 ▶); cell refinement: *CrystalClear*; data reduction: *CrystalClear*; program(s) used to solve structure: *SHELXS97* (Sheldrick, 2008 ▶); program(s) used to refine structure: *SHELXL97* (Sheldrick, 2008 ▶); molecular graphics: *SHELXTL* (Sheldrick, 2008 ▶); software used to prepare material for publication: *PRPKAPPA* (Ferguson, 1999 ▶).

## Supplementary Material

Crystal structure: contains datablocks I, global. DOI: 10.1107/S1600536809036101/er2072sup1.cif

Structure factors: contains datablocks I. DOI: 10.1107/S1600536809036101/er2072Isup2.hkl

Additional supplementary materials:  crystallographic information; 3D view; checkCIF report

## Figures and Tables

**Table 1:** Hydrogen-bond geometry (Å, °)

*D*—H⋯*A*	*D*—H	H⋯*A*	*D*⋯*A*	*D*—H⋯*A*
N1—H1⋯O1^i^	0.86	2.12	2.962 (2)	166

Symmetry code: (i) 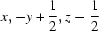.

**Fig. 1.**
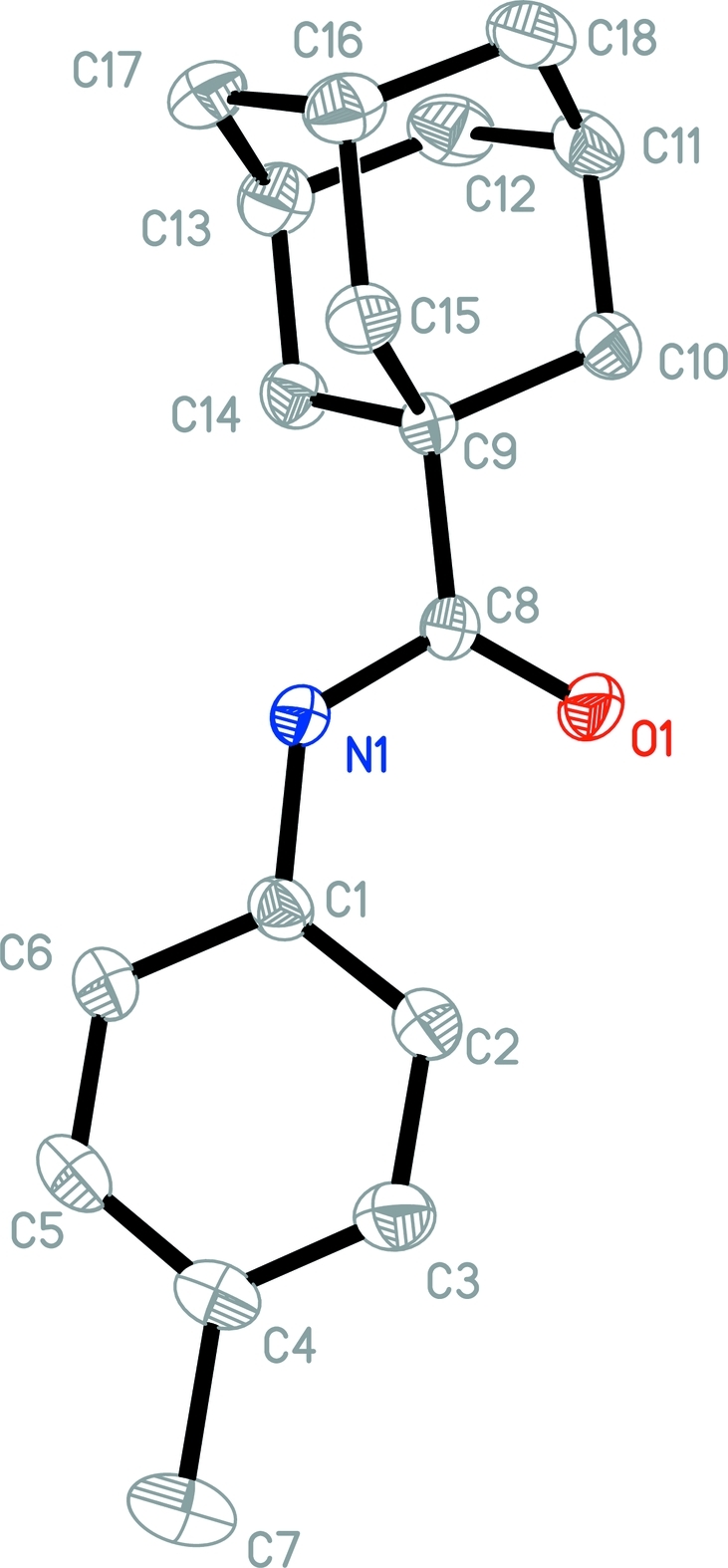
The molecular structure of the title compound, with the atomic numbering scheme. Displacement ellipsoids are drawn at the 30% probability level, and all H atoms have been omitted for clarity.

**Fig. 2.**
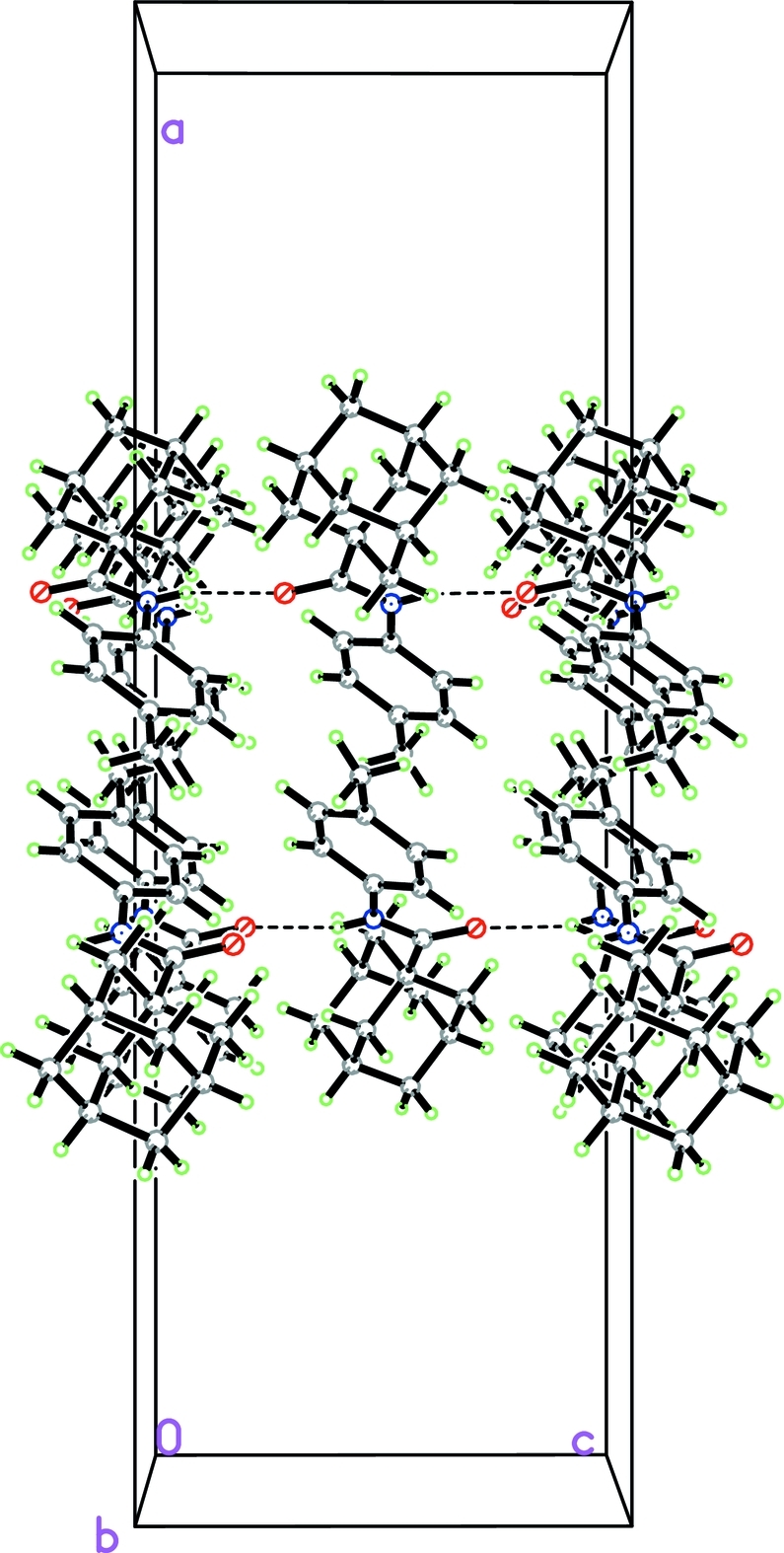
A view of the packing of the title compound, stacking along the b axis. Dashed lines indicate hydrogen bonds.

## supplementary crystallographic information

### Comment

The unique structure of adamantane and the pharmaccutical effects of adamantane-containing agents on virus (Davis *et al.*, 1964) have attracted many chemists and pharmacologists to do considerable work on the syntheses of adamantane derivatives (Fort *et al.*, 1964). The crystal structure of the title compound (I) is reported herein.

The molecular structure of compound (I), C_18_H_23_ON, is shown in Figure 1. All bond lengths and bond correspond to the geometry parameters expected for atom types and the type of hybridization (Allen *et al.*, 1987). The bonds to nitrogen of the title amide, Fig. 1, the torsion angles of O1—C8—N1—C1 and C9—C8—N1—C1 are 1.70 (3)° and 178.59 (18)°, respectively. The C8—N1 bond has considerable double-bond characer, at 1.349 (2) Å, is substantially shorter than the normal C—N single-bond distance observed in amines. In the crystal of (I), the intermolecular N_1_—H···O_1_ H-bonds linked molecules to chains along the *c* axis (Fig.2). And the N_1_—H···O_1_ bond length is 2.962 (2) Å.

### Experimental

A solution of freshly prepared 1-adamantane carbonyl chloride (1 mmol, prepared by refluxing 1-adamantane carboxylic acid with 3*M* excess of SOCl_2_) in dry CH_2_Cl_2_ was added dropwise to a well stirred and ice-cooled solution of *p*-toluidine (1 mmol) and triethylamine (2 mmol) in the same solvent. After 24 h of stirring at room temperature, the solvents were removed *in vacuo* and the residue was recrystallized from methanol. Colorless single crystals of the title compound suitable for X-ray diffraction analysis were obtained then and the yield was 80% (Isabella *et al.* 1997; Tadashi *et al.*, 1969).

### Refinement

Positional parameters of all the H atoms were calculated geometrically and were allowed to ride on the C atoms to which they are bonded, with *U*_iso_(H) = 1.2*U*_eq_(C).

### Crystal data

**Table:**

C_18_H_23_NO	*F*(000) = 1168
*M**_r_* = 269.37	*D*_x_ = 1.188 Mg m^−^^3^
Orthorhombic, *P**c**c**n*	Mo *K*α radiation, λ = 0.71073 Å
Hall symbol: -P 2ab 2ac	Cell parameters from 4945 reflections
*a* = 30.708 (7) Å	θ = 2.5–27.5°
*b* = 9.7927 (2) Å	µ = 0.07 mm^−^^1^
*c* = 10.0203 (6) Å	*T* = 298 K
*V* = 3013.2 (7) Å^3^	Block, colourless
*Z* = 8	0.50 × 0.30 × 0.30 mm

### Data collection

**Table:**

Rigaku SCXmini diffractometer	3443 independent reflections
Radiation source: fine-focus sealed tube	2652 reflections with *I* > 2σ(*I*)
graphite	*R*_int_ = 0.080
Detector resolution: 13.6612 pixels mm^-1^	θ_max_ = 27.5°, θ_min_ = 2.7°
CCD Profile fitting scans	*h* = −39→39
Absorption correction: multi-scan (*CrystalClear*; Rigaku, 2005)	*k* = −12→12
*T*_min_ = 0.964, *T*_max_ = 0.978	*l* = −12→12
27405 measured reflections	

### Refinement

**Table:**

Refinement on *F*^2^	Primary atom site location: structure-invariant direct methods
Least-squares matrix: full	Secondary atom site location: difference Fourier map
*R*[*F*^2^ > 2σ(*F*^2^)] = 0.073	Hydrogen site location: inferred from neighbouring sites
*wR*(*F*^2^) = 0.174	H-atom parameters constrained
*S* = 1.16	*w* = 1/[σ^2^(*F*_o_^2^) + (0.0692*P*)^2^ + 0.7031*P*] where *P* = (*F*_o_^2^ + 2*F*_c_^2^)/3
3443 reflections	(Δ/σ)_max_ = 0.001
182 parameters	Δρ_max_ = 0.20 e Å^−^^3^
0 restraints	Δρ_min_ = −0.15 e Å^−^^3^

### Special details

**Table:**

Geometry. All e.s.d.'s (except the e.s.d. in the dihedral angle between two l.s. planes) are estimated using the full covariance matrix. The cell e.s.d.'s are taken into account individually in the estimation of e.s.d.'s in distances, angles and torsion angles; correlations between e.s.d.'s in cell parameters are only used when they are defined by crystal symmetry. An approximate (isotropic) treatment of cell e.s.d.'s is used for estimating e.s.d.'s involving l.s. planes.
Refinement. Refinement of *F*^2^ against ALL reflections. The weighted *R*-factor *wR* and goodness of fit *S* are based on *F*^2^, conventional *R*-factors *R* are based on *F*, with *F* set to zero for negative *F*^2^. The threshold expression of *F*^2^ > σ(*F*^2^) is used only for calculating *R*-factors(gt) *etc*. and is not relevant to the choice of reflections for refinement. *R*-factors based on *F*^2^ are statistically about twice as large as those based on *F*, and *R*- factors based on ALL data will be even larger.

### Fractional atomic coordinates and isotropic or equivalent isotropic displacement parameters (Å^2^)

**Table:**

	*x*	*y*	*z*	*U*_iso_*/*U*_eq_	
C1	0.41659 (6)	0.06223 (19)	−0.01785 (19)	0.0377 (4)	
C2	0.41516 (7)	−0.0339 (2)	0.0824 (2)	0.0487 (5)	
H2	0.3978	−0.0195	0.1570	0.058*	
C3	0.43961 (8)	−0.1518 (2)	0.0718 (2)	0.0531 (6)	
H3	0.4382	−0.2159	0.1401	0.064*	
C4	0.46586 (7)	−0.1775 (2)	−0.0360 (2)	0.0497 (5)	
C5	0.46711 (7)	−0.0805 (2)	−0.1354 (2)	0.0531 (6)	
H5	0.4846	−0.0952	−0.2097	0.064*	
C6	0.44298 (7)	0.0383 (2)	−0.1273 (2)	0.0485 (5)	
H6	0.4445	0.1024	−0.1956	0.058*	
C7	0.49221 (9)	−0.3075 (3)	−0.0462 (3)	0.0744 (8)	
H7A	0.5182	−0.2903	−0.0967	0.112*	
H7B	0.4752	−0.3765	−0.0901	0.112*	
H7C	0.4999	−0.3382	0.0417	0.112*	
C8	0.37650 (6)	0.2542 (2)	0.08852 (18)	0.0364 (4)	
C9	0.34923 (6)	0.38031 (19)	0.05641 (18)	0.0351 (4)	
C10	0.33115 (7)	0.4418 (2)	0.1861 (2)	0.0472 (5)	
H10A	0.3550	0.4667	0.2446	0.057*	
H10B	0.3133	0.3746	0.2317	0.057*	
C11	0.30364 (8)	0.5690 (2)	0.1545 (2)	0.0534 (6)	
H11	0.2923	0.6076	0.2378	0.064*	
C12	0.33198 (9)	0.6739 (2)	0.0850 (3)	0.0618 (7)	
H12A	0.3561	0.6990	0.1424	0.074*	
H12B	0.3151	0.7555	0.0667	0.074*	
C13	0.34927 (8)	0.6146 (2)	−0.0453 (2)	0.0549 (6)	
H13	0.3671	0.6831	−0.0910	0.066*	
C14	0.37712 (7)	0.4883 (2)	−0.0142 (2)	0.0446 (5)	
H14A	0.3888	0.4509	−0.0964	0.053*	
H14B	0.4014	0.5140	0.0425	0.053*	
C15	0.31066 (7)	0.3420 (2)	−0.0341 (2)	0.0437 (5)	
H15A	0.3214	0.3033	−0.1169	0.052*	
H15B	0.2928	0.2737	0.0098	0.052*	
C16	0.28321 (7)	0.4687 (2)	−0.0641 (2)	0.0535 (6)	
H16	0.2587	0.4435	−0.1217	0.064*	
C17	0.31163 (8)	0.5726 (3)	−0.1349 (2)	0.0591 (6)	
H17A	0.2944	0.6523	−0.1578	0.071*	
H17B	0.3229	0.5335	−0.2169	0.071*	
C18	0.26607 (8)	0.5283 (3)	0.0655 (3)	0.0599 (6)	
H18A	0.2482	0.6076	0.0466	0.072*	
H18B	0.2481	0.4612	0.1108	0.072*	
N1	0.39146 (6)	0.18381 (17)	−0.01773 (15)	0.0414 (4)	
H1	0.3850	0.2166	−0.0948	0.050*	
O1	0.38450 (5)	0.21905 (16)	0.20311 (13)	0.0529 (4)	

### Atomic displacement parameters (Å^2^)

**Table:**

	*U*^11^	*U*^22^	*U*^33^	*U*^12^	*U*^13^	*U*^23^
C1	0.0387 (10)	0.0392 (10)	0.0352 (10)	0.0041 (8)	−0.0014 (8)	−0.0063 (8)
C2	0.0507 (12)	0.0489 (12)	0.0466 (12)	0.0075 (10)	0.0105 (10)	0.0025 (10)
C3	0.0563 (13)	0.0424 (12)	0.0605 (14)	0.0078 (10)	0.0049 (11)	0.0054 (10)
C4	0.0426 (11)	0.0435 (12)	0.0630 (14)	0.0054 (10)	−0.0001 (11)	−0.0114 (10)
C5	0.0505 (12)	0.0568 (14)	0.0521 (13)	0.0072 (11)	0.0102 (10)	−0.0147 (11)
C6	0.0549 (13)	0.0503 (12)	0.0401 (11)	0.0064 (10)	0.0073 (9)	−0.0032 (9)
C7	0.0662 (16)	0.0551 (15)	0.102 (2)	0.0201 (13)	0.0048 (16)	−0.0105 (15)
C8	0.0393 (10)	0.0396 (10)	0.0301 (9)	0.0009 (8)	−0.0011 (8)	−0.0010 (8)
C9	0.0373 (10)	0.0367 (10)	0.0314 (9)	0.0023 (8)	0.0015 (8)	−0.0010 (8)
C10	0.0554 (13)	0.0460 (12)	0.0401 (11)	0.0084 (10)	0.0054 (9)	−0.0034 (9)
C11	0.0580 (13)	0.0476 (13)	0.0546 (13)	0.0119 (11)	0.0107 (11)	−0.0057 (10)
C12	0.0654 (15)	0.0386 (12)	0.0813 (18)	0.0055 (11)	0.0027 (13)	−0.0045 (12)
C13	0.0574 (13)	0.0424 (12)	0.0650 (15)	−0.0040 (10)	0.0069 (12)	0.0133 (11)
C14	0.0436 (11)	0.0420 (11)	0.0480 (11)	−0.0012 (9)	0.0047 (9)	0.0015 (9)
C15	0.0420 (11)	0.0435 (11)	0.0455 (11)	−0.0007 (9)	−0.0027 (9)	−0.0001 (9)
C16	0.0459 (12)	0.0566 (14)	0.0580 (13)	0.0049 (11)	−0.0076 (10)	0.0047 (11)
C17	0.0641 (15)	0.0552 (14)	0.0580 (14)	0.0144 (12)	−0.0006 (12)	0.0184 (12)
C18	0.0455 (12)	0.0543 (14)	0.0799 (17)	0.0116 (11)	0.0081 (12)	0.0055 (13)
N1	0.0521 (10)	0.0437 (9)	0.0284 (8)	0.0141 (8)	0.0000 (7)	0.0007 (7)
O1	0.0778 (11)	0.0511 (9)	0.0297 (7)	0.0213 (8)	−0.0035 (7)	−0.0015 (6)

### Geometric parameters (Å, °)

**Table:**

C1—C2	1.377 (3)	C10—H10B	0.9700
C1—C6	1.383 (3)	C11—C18	1.512 (3)
C1—N1	1.419 (2)	C11—C12	1.516 (3)
C2—C3	1.381 (3)	C11—H11	0.9800
C2—H2	0.9300	C12—C13	1.525 (3)
C3—C4	1.371 (3)	C12—H12A	0.9700
C3—H3	0.9300	C12—H12B	0.9700
C4—C5	1.377 (3)	C13—C17	1.520 (3)
C4—C7	1.512 (3)	C13—C14	1.536 (3)
C5—C6	1.382 (3)	C13—H13	0.9800
C5—H5	0.9300	C14—H14A	0.9700
C6—H6	0.9300	C14—H14B	0.9700
C7—H7A	0.9600	C15—C16	1.530 (3)
C7—H7B	0.9600	C15—H15A	0.9700
C7—H7C	0.9600	C15—H15B	0.9700
C8—O1	1.224 (2)	C16—C17	1.517 (3)
C8—N1	1.349 (2)	C16—C18	1.518 (3)
C8—C9	1.526 (3)	C16—H16	0.9800
C9—C14	1.534 (3)	C17—H17A	0.9700
C9—C10	1.536 (3)	C17—H17B	0.9700
C9—C15	1.538 (3)	C18—H18A	0.9700
C10—C11	1.537 (3)	C18—H18B	0.9700
C10—H10A	0.9700	N1—H1	0.8600
			
C2—C1—C6	118.76 (19)	C11—C12—C13	109.58 (19)
C2—C1—N1	123.74 (17)	C11—C12—H12A	109.8
C6—C1—N1	117.48 (18)	C13—C12—H12A	109.8
C1—C2—C3	119.8 (2)	C11—C12—H12B	109.8
C1—C2—H2	120.1	C13—C12—H12B	109.8
C3—C2—H2	120.1	H12A—C12—H12B	108.2
C4—C3—C2	122.3 (2)	C17—C13—C12	110.1 (2)
C4—C3—H3	118.9	C17—C13—C14	109.01 (19)
C2—C3—H3	118.9	C12—C13—C14	109.09 (19)
C3—C4—C5	117.4 (2)	C17—C13—H13	109.5
C3—C4—C7	121.5 (2)	C12—C13—H13	109.5
C5—C4—C7	121.1 (2)	C14—C13—H13	109.5
C4—C5—C6	121.6 (2)	C9—C14—C13	109.75 (17)
C4—C5—H5	119.2	C9—C14—H14A	109.7
C6—C5—H5	119.2	C13—C14—H14A	109.7
C5—C6—C1	120.2 (2)	C9—C14—H14B	109.7
C5—C6—H6	119.9	C13—C14—H14B	109.7
C1—C6—H6	119.9	H14A—C14—H14B	108.2
C4—C7—H7A	109.5	C16—C15—C9	109.99 (17)
C4—C7—H7B	109.5	C16—C15—H15A	109.7
H7A—C7—H7B	109.5	C9—C15—H15A	109.7
C4—C7—H7C	109.5	C16—C15—H15B	109.7
H7A—C7—H7C	109.5	C9—C15—H15B	109.7
H7B—C7—H7C	109.5	H15A—C15—H15B	108.2
O1—C8—N1	121.90 (18)	C17—C16—C18	110.0 (2)
O1—C8—C9	122.38 (17)	C17—C16—C15	108.61 (18)
N1—C8—C9	115.72 (15)	C18—C16—C15	109.57 (19)
C8—C9—C14	110.41 (15)	C17—C16—H16	109.5
C8—C9—C10	109.71 (15)	C18—C16—H16	109.5
C14—C9—C10	108.77 (16)	C15—C16—H16	109.5
C8—C9—C15	110.43 (16)	C16—C17—C13	110.00 (19)
C14—C9—C15	109.04 (16)	C16—C17—H17A	109.7
C10—C9—C15	108.43 (16)	C13—C17—H17A	109.7
C9—C10—C11	110.01 (17)	C16—C17—H17B	109.7
C9—C10—H10A	109.7	C13—C17—H17B	109.7
C11—C10—H10A	109.7	H17A—C17—H17B	108.2
C9—C10—H10B	109.7	C11—C18—C16	109.94 (19)
C11—C10—H10B	109.7	C11—C18—H18A	109.7
H10A—C10—H10B	108.2	C16—C18—H18A	109.7
C18—C11—C12	110.2 (2)	C11—C18—H18B	109.7
C18—C11—C10	109.11 (18)	C16—C18—H18B	109.7
C12—C11—C10	109.15 (18)	H18A—C18—H18B	108.2
C18—C11—H11	109.4	C8—N1—C1	127.93 (16)
C12—C11—H11	109.4	C8—N1—H1	116.0
C10—C11—H11	109.4	C1—N1—H1	116.0
			
C6—C1—C2—C3	−0.4 (3)	C11—C12—C13—C14	−61.2 (2)
N1—C1—C2—C3	177.7 (2)	C8—C9—C14—C13	−179.79 (16)
C1—C2—C3—C4	0.3 (4)	C10—C9—C14—C13	−59.4 (2)
C2—C3—C4—C5	−0.1 (4)	C15—C9—C14—C13	58.7 (2)
C2—C3—C4—C7	−179.7 (2)	C17—C13—C14—C9	−59.7 (2)
C3—C4—C5—C6	0.1 (4)	C12—C13—C14—C9	60.5 (2)
C7—C4—C5—C6	179.6 (2)	C8—C9—C15—C16	179.22 (16)
C4—C5—C6—C1	−0.2 (3)	C14—C9—C15—C16	−59.3 (2)
C2—C1—C6—C5	0.4 (3)	C10—C9—C15—C16	59.0 (2)
N1—C1—C6—C5	−177.82 (19)	C9—C15—C16—C17	60.4 (2)
O1—C8—C9—C14	114.5 (2)	C9—C15—C16—C18	−59.8 (2)
N1—C8—C9—C14	−65.3 (2)	C18—C16—C17—C13	58.4 (2)
O1—C8—C9—C10	−5.4 (3)	C15—C16—C17—C13	−61.5 (3)
N1—C8—C9—C10	174.87 (17)	C12—C13—C17—C16	−58.3 (3)
O1—C8—C9—C15	−124.8 (2)	C14—C13—C17—C16	61.3 (2)
N1—C8—C9—C15	55.4 (2)	C12—C11—C18—C16	59.4 (2)
C8—C9—C10—C11	180.00 (17)	C10—C11—C18—C16	−60.5 (2)
C14—C9—C10—C11	59.1 (2)	C17—C16—C18—C11	−58.9 (2)
C15—C9—C10—C11	−59.3 (2)	C15—C16—C18—C11	60.4 (2)
C9—C10—C11—C18	60.4 (2)	O1—C8—N1—C1	1.7 (3)
C9—C10—C11—C12	−60.1 (2)	C9—C8—N1—C1	−178.59 (18)
C18—C11—C12—C13	−59.0 (2)	C2—C1—N1—C8	28.1 (3)
C10—C11—C12—C13	60.8 (3)	C6—C1—N1—C8	−153.8 (2)
C11—C12—C13—C17	58.4 (3)		

### Hydrogen-bond geometry (Å, °)

**Table:**

*D*—H···*A*	*D*—H	H···*A*	*D*···*A*	*D*—H···*A*
N1—H1···O1^i^	0.86	2.12	2.962 (2)	166

Symmetry codes: (i) *x*, −*y*+1/2, *z*−1/2.
